# Importance of leptin signaling and signal transducer and activator of transcription-3 activation in mediating the cardiac hypertrophy associated with obesity

**DOI:** 10.1186/1479-5876-11-170

**Published:** 2013-07-11

**Authors:** Maren Leifheit-Nestler, Nana-Maria Wagner, Rajinikanth Gogiraju, Michael Didié, Stavros Konstantinides, Gerd Hasenfuss, Katrin Schäfer

**Affiliations:** 1 Department of Cardiology and Pulmonary Medicine, Heart Research Center, Georg August University Medicine Goettingen, Robert Koch Strasse 40, D-37075, Göttingen, Germany; 2 Current address: Department of Pediatric Kidney, Liver and Metabolic Diseases, Hannover Medical School, Hannover, Germany; 3 Current address: Clinic for Anesthesiology and Intensive Care Medicine, University Medicine Rostock, Rostock, Germany; 4 Department of Pharmacology, Georg August University Medicine Goettingen, Goettingen, Germany; 5 Current address: Center for Thrombosis and Hemostasis, University Medicine Mainz, Mainz, Germany

**Keywords:** Heart, Hypertrophy, Leptin, Obesity, Signal transduction, STAT3

## Abstract

**Background:**

The adipokine leptin and its receptor are expressed in the heart, and leptin has been shown to promote cardiomyocyte hypertrophy *in vitro*. Obesity is associated with hyperleptinemia and hypothalamic leptin resistance as well as an increased risk to develop cardiac hypertrophy and heart failure. However, the role of cardiac leptin signaling in mediating the cardiomyopathy associated with increased body weight is unclear, in particular, whether it develops subsequently to cardiac leptin resistance or overactivation of hypertrophic signaling pathways via elevated leptin levels.

**Methods:**

The cardiac phenotype of high-fat diet (HFD)-induced obese wildtype (WT) mice was examined and compared to age-matched genetically obese leptin receptor (LepR)-deficient (LepR^db/db^) or lean WT mice. To study the role of leptin-mediated STAT3 activation during obesity-induced cardiac remodeling, mice in which tyrosine residue 1138 within LepR had been replaced with a serine (LepR^S1138^) were also analyzed.

**Results:**

Obesity was associated with hyperleptinemia and elevated cardiac leptin expression in both diet-induced and genetically obese mice. Enhanced LepR and STAT3 phosphorylation levels were detected in hearts of obese WT mice, but not in those with LepR mutations. Moreover, exogenous leptin continued to induce cardiac STAT3 activation in diet-induced obese mice. Although echocardiography revealed signs of cardiac hypertrophy in all obese mice, the increase in left ventricular (LV) mass and diameter was significantly more pronounced in LepR^S1138^ animals. LepR^S1138^ mice also exhibited an increased activation of signaling proteins downstream of LepR, including Jak2 (1.8-fold), Src kinase (1.7-fold), protein kinase B (1.3-fold) or C (1.6-fold). Histological analysis of hearts revealed that the inability of leptin to activate STAT3 in LepR^db/db^ and LepR^S1138^ mice was associated with reduced cardiac angiogenesis as well as increased apoptosis and fibrosis.

**Conclusions:**

Our findings suggest that hearts from obese mice continue to respond to elevated circulating or cardiac leptin, which may mediate cardioprotection via LepR-induced STAT3 activation, whereas signals distinct from LepR-Tyr1138 promote cardiac hypertrophy. On the other hand, the presence of cardiac hypertrophy in obese mice with complete LepR signal disruption indicates that additional pathways also play a role.

## Background

Obesity is frequently associated with elevated circulating leptin levels [1] and an increased risk to develop cardiac hypertrophy [2,3] or heart failure [4]. Clinical studies demonstrated a positive correlation between serum leptin levels and left ventricular (LV) mass or wall thickness [5,6], independent of blood pressure levels, suggesting a direct role for leptin in the pathogenesis of obesity-associated cardiomyopathy. Furthermore, leptin was shown to promote hypertrophy of isolated rat or human ventricular cardiomyocytes [7,8], and this effect could be prevented using neutralizing antibodies [9].

Cardiac hypertrophy also develops in obese rodents fed high-fat diet (HFD) [10,11], and studies in mice with (functional) leptin deficiency suggested that the cardiac hypertrophy developing in states of chronic hyperleptinemia may result from the inability to transduce anti-hypertrophic and/or cardioprotective effects of the adipokine [12,13]. While the effects of leptin on cell shortening and intracellular Ca^2+^ transients were found to be abrogated in cardiomyocytes isolated from HFD-fed obese rats [14], others reported a preserved signal transduction in response to leptin in hyperleptinemic obese mice [15,16] or rats [17]. Thus, the role of the adipokine in mediating cardiac hypertrophy, in particular in the presence of elevated systemic leptin levels, and the possible existence of a cardiac leptin resistance in obesity remains unclear.

The leptin receptor (LepR) belongs to the family of cytokine type I receptors known to signal via activation of Janus kinase (Jak)-2 and signal transducer and activator of transcription (STAT)-3 [18]. Analysis of cardiomyocytes *ex vivo* revealed that leptin promotes hypertrophy via activation of p38 and p42/44 MAP kinases as well as protein kinase B (Akt) [19,20]. On the other hand, it is unknown whether STAT3 activation downstream of LepR is required to transmit the cardiac effects of leptin and whether it may be involved in mediating protective (i.e. anti-apoptotic, anti-fibrotic or pro-angiogenic) signals, as previously reported in mice with cardiomyocyte-specific STAT3 deletion [21,22].

In this study, we examined the cardiac phenotype of diet-induced (i.e. with hypothalamic leptin resistance) and genetically obese (i.e. with systemic leptin receptor deficiency) hyperleptinemic mice, developing with age or after continuous β-adrenergic stimulation. Moreover, we determined the importance of leptin-mediated STAT3 activation for the development of cardiac hypertrophy in obesity by analyzing mice with targeted mutation of the STAT3 binding site within LepR.

## Methods

### Animals

C57Bl6/J leptin receptor-deficient db/db (LepR^db/db^; BKS.Cg Lepr^db^/Lepr^db^) mice and C57Bl6/J wildtype (WT) controls were obtained from Harlan Winkelmann, Germany. Mice heterozygous mutant for the LepR^S1138^ allele (on the congenic B6.129/J background; 98- > 99% homozygous for C57Bl/6; [23]) were obtained from Professor Martin Myers (University of Michigan Medical School, Ann Arbor, USA) and bred at the animal facility of the University of Goettingen, Germany, to generate homozygous mutant obese LepR^S1138^ mice. Age- and gender-matched WT (LepR^+/+^) and heterozygous (LepR^S/+^) littermates were used as controls. To induce obesity, 3 months-old mice were switched to high-fat diet (HFD; D12451) for 4 months, while controls were maintained on normal rodent chow (D12450B; both Research Diets Inc.). The composition of both diets is shown in Additional file 1: Table S1. To examine the cardiac response to hypertrophic stimuli other than leptin, osmotic minipumps (Alzet®; model 2002; Charles River Laboratories) were filled with isoprenaline hydrochloride (Sigma; 20 mg/kg body weight [BW] per day) and implanted for 14 days under the dorsal skinfold of 2 months-old, 2% isoflurane anesthetized mice. At the time of tissue harvest, mice were weighed followed by intraperitoneal anesthesia with a mixture of 2% xylazine (6 mg/kg BW) and 10% ketamine hydrochloride (100 mg/kg BW), and blood was drawn by cardiac puncture. Hearts were rapidly excised, the atria removed and ventricles immediately processed for protein isolation or cryoembedding, respectively. All animal care and experimental procedures had been approved by the institutional Animal Research Committee and complied with national guidelines for the care and use of laboratory animals.

### Serum analysis

Freshly drawn blood was allowed to clot at room temperature (RT) for 30 min, followed by centrifugation for 10 min at 3,000 rpm. The supernatant was stored at -80°C pending analysis of serum leptin levels using specific enzyme-linked immunoassays (ELISA; R&D Systems).

### Echocardiography

Echocardiography was performed by a blinded examiner at the day before tissue harvest in mice under 1.5% isoflurane anesthesia using the VisualSonics Vevo 2100 system (Visualsonics) equipped with a 30 MHz center frequency ultrasound transducer, as previously described [24]. M-mode echocardiographical recordings were used to determine the end-diastolic and end-systolic LV diameter (EDD and ESD, respectively) and the ventricular wall thickness (WTh), corresponding to the mean of the anterior and posterior WTh. LV mass was calculated using the formula: 1.055 × ([AWTh + EDD + PWTh]^3^ - EDD^3^). Fractional shortening (FS) was calculated as (EDD - ESD)/EDD × 100. B-mode echocardiography images were used to calculate the heart weight, using the equation: 1.05 × (5/6) × ((Epi_syst_ × (L_syst_ + ((AWTh_syst_ + PWTh_syst_)/2))) - (Area_syst_ × L_syst_)).

### Histology and immunohistochemistry

Histochemical analyses were performed on 5 μm-thick frozen cross sections through the LV. For each mouse, 4 sections (approx. 500 μm apart) and 4 randomly selected viewing fields (at 200-fold magnification) per section were analyzed and findings averaged. Cardiac fibrosis was determined after overnight incubation in Bouin’s fixative followed by Masson’s trichrome (MTC) stain. Monoclonal rat antibodies against mouse CD31 (Santa Cruz Biotechnology) were used to detect endothelial cells [24,25]. Their number was manually counted by a person blinded to the mouse genotype and expressed per mm^2^ or cardiomyocyte, respectively. Single cardiomyocytes were visualized by incubation with fluorescein-labeled wheat germ agglutinin (WGA; Molecular Probes), followed by determination of the cardiomyocyte cross-sectional area (CSA) using image analysis software (Image ProPlus). Per cross section, at least 10 randomly selected cardiomyocytes were evaluated and results averaged. Apoptosis was analyzed using the ‘*In Situ* Cell Death Detection kit’ (Roche). Cell nuclei were visualized using 4′,6-diamidino-2-phenylindole (DAPI; Sigma).

### Immunoprecipitation and western blot analysis

Frozen whole heart tissue (after removal of both atria) was pulverized on liquid nitrogen and resuspended in 500 μL lysis buffer (1% Triton-X 100, 150 mM NaCl, 50 mM TRIS, 5 mM EDTA, pH 7.5) containing fresh protease (4 μg/mL of each aprotinin, leupeptin and pepstatin A, 1 mM PMSF) and phosphatase (20 mM NaF and 1 mM Na_3_VO_4_) inhibitors. After incubation for 20 min on ice, lysates were cleared by centrifugation at 13,000 rpm for 10 min at 4°C. Equal amounts (50 μg) of protein were fractionated by SDS polyacrylamide gel electrophoresis together with molecular weight standards and transferred to nitrocellulose membranes (Protran®, Whatman). Membranes were blocked in 1% bovine serum albumine (in TBS, containing 0.1% Tween-20) prior to incubation with antibodies against phosphorylated (p)-Akt (S473) and total Akt, p-Jak2 (Y1007/1008) and total Jak2, p-p38 (T180/Y182) and total p38, p-p42/44 (T202/Y204) and total p42/44, p-Src (Y416) and total Src, p-STAT3 (Y705) and total STAT3, or p-PKC (pan), respectively (all Cell Signaling Technologies), or against leptin (R&D Systems) and GAPDH (Biotrend), respectively. Protein bands were visualized using HRP-conjugated secondary antibodies (Amersham Biosciences), followed by detection with SuperSignal® West Pico Substrate (Pierce). For the analysis of LepR phosphorylation, 100 μg total heart tissue lysates were immunoprecipitated under rotation at 4°C with 2 μg anti-LepR antibody (against an internal domain present in the short and long isoforms of murine LepR; Santa Cruz Biotechnology) plus 50 μL nProtein A Sepharose™ 4 Fast Flow beads (GE Healthcare) followed by detection of phosphorylated tyrosines (p-Tyr [PY20]; Santa Cruz Biotechnology) or LepR. For the analysis of STAT3 phosphorylation in response to acute elevations of circulating leptin, mice were fasted overnight, injected with recombinant murine leptin (1 mg/kg BW i.p.) and hearts harvested 30 min later. Protein bands were quantified by densitometry and results expressed as % of total protein (after normalization for GAPDH expression).

### Statistical analysis

Quantitative data are presented as mean ± standard error of the mean (SEM). Normal data distribution was tested using the D’Agostino & Pearson omnibus normality test. When three or more groups were compared, ANOVA was employed, if samples were normally distributed, or Kruskal-Wallis test, if not. For *post-hoc* comparisons, ANOVA was followed by Bonferroni’s and Kruskal-Wallis by Dunn’s multiple comparison test. Differences before and after isoprenaline infusion were tested using Student’s *t*-test for paired means. Statistical significance was assumed when P reached a value less than 0.05. All statistical analyses were performed using GraphPad PRISM software, version 4.01 (GraphPad Software Inc).

## Results

**Figure 1:**
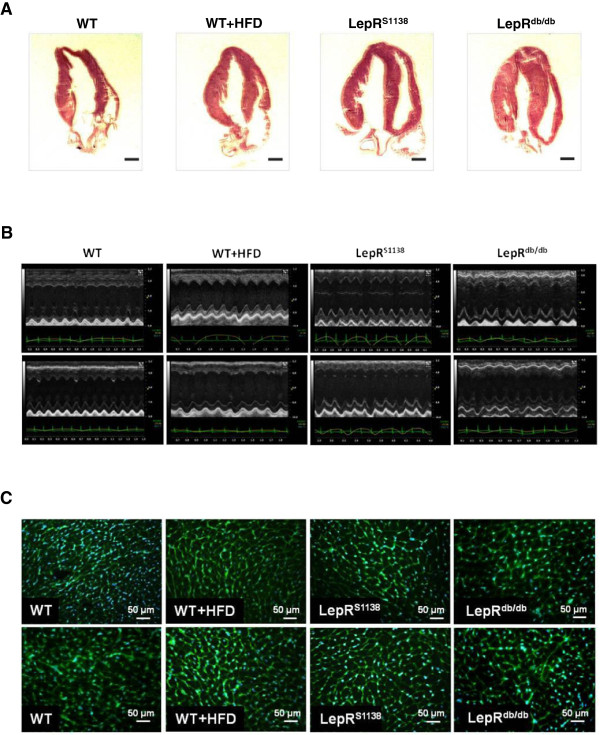
**Cardiac phenotype of lean and obese WT, WT + HFD, LepR**^**S1138 **^**and LepR**^**db/db **^**mice. (A)** Representative H&E-stained longitudinal sections through hearts of 7 months-old mice are shown. Magnification, ×10. **(B)** Representative M-mode echocardiographic recordings. **(C)** Representative images of wheat germ agglutinin (WGA)-stained myocardial cross sections. The mean cardiomyocyte cross-sectional areas are given in Table 1.

Clinical and experimental studies revealed that obesity is associated with LV hypertrophy [10,11], an important risk factor for the development of heart failure. As shown in Tables 1 and 2, WT mice fed HFD for 4 months (WT + HFD; mean body weight [BW], 44±1.9 g) to induce obesity exhibited a non-significant trend towards an increased mean heart weight, LV mass and WTh compared to age-matched lean controls fed normal chow (BW, 29±1.0 g). Marked LV hypertrophy was observed in 7 months-old obese LepR^db/db^ mice (Table 1 and 2), consistent with a previous report [12]. Longitudinal sections through hearts of WT, WT + HFD and LepR^db/db^ mice are shown in Figure 1A, representative M-mode echocardiography recordings in Figure 1B and cardiac cross-sections after WGA staining to delineate cardiomyocyte borders in Figure 1C. Of note, adiposity in mice with LepR deficiency was more pronounced compared to age-matched WT + HFD mice (Table 1; P < 0.001), in which obesity develops as result of hypothalamic resistance to chronically elevated leptin levels [26].

**Table 1:** Body, visceral fat and heart weights in 7 months-old mice

	**WT**	**WT + HFD**	**LepR**^**S1138**^	**LepR**^**db/db**^
**n**	**25**	**12**	**30**	**30**
**body weight** (g)	29 ± 1.0	44 ± 1.9***	54 ± 1.1*** ###	55 ± 1.5*** ###
**VAT** (mg)	0.6 ± 0.1	1.7 ± 0.3*	3.3 ± 0.3*** ###	2.7 ± 0.2*** #
**serum leptin** (ng/mL)	12 ± 1.3	60 ± 6.0***	67 ± 5.9*** §§§	118 ± 6.8*** ###
**heart weight** (mg)	135 ± 3.6	147 ± 5.8	212 ± 11*** ### §§§	151 ± 5.2
**normalized heart weight** (mg/mm TL)	7.5 ± 0.2	8.2 ± 0.3	12 ± 0.6*** ### §§§	8.9 ± 0.2
**normalized heart weight** (mg/g BW)	4.8 ± 0.1	3.4 ± 0.2***	3.9 ± 0.2*** §§§	2.8 ± 0.1***
**CSA** (μm^2^)	148 ± 4.6	161 ± 6.4	183 ± 9.9**	165 ± 6.5

*P < 0.05, **P < 0.01 and ***P < 0.001 vs. WT mice; #P < 0.05 and ###P < 0.001 vs. WT mice fed HFD; §§§P < 0.001 for the difference between LepR^S1138^ and LepR^db/db^ mice. Abbreviations: *BW*, body weight; *CSA*, cross-sectional area; *HFD*, high-fat diet; *TL*, tibia length; *VAT*, visceral adipose tissue.

**Table 2:** Echocardiographic parameter in 7 months-old mice

	**WT**	**WT + HFD**	**LepR**^**S1138**^	**LepR**^**db/db**^
**n**	**25**	**12**	**30**	**30**
**heart rate** (bpm)	463 ± 6.5	481 ± 6.7	459 ± 10	460 ± 11
**WTh** (mm)	0.70 ± 0.01	0.75 ± 0.02	0.85 ± 0.02*** #	0.81 ± 0.02**
**ESD** (mm)	3.2 ± 0.1	3.5 ± 0.1*	3.2 ± 0.1# §§	3.0 ± 0.1* ###
**EDD** (mm)	4.3 ± 0.1	4.5 ± 0.1	4.6 ± 0.1** §§	4.3 ± 0.03
**LVM** (mg)	116 ± 4.2	132 ± 5.8	166 ± 7.2*** # §§	137 ± 5.5**
**HW** (mg)	118 ± 3.3	121 ± 3.6	135 ± 4.6** §§§	108 ± 2.0
**FS** (%)	26 ± 0.7	22 ± 0.8	31 ± 1.1* ###	31 ± 0.9** ###

*P < 0.05, **P < 0.01 and ***P < 0.001 vs. WT mice; #P < 0.05 and ###P < 0.001 vs. WT mice fed HFD; §§P < 0.01 and §§§P < 0.001 for the difference between LepR^S1138^ and LepR^db/db^ mice. Abbreviations: bpm, beats per minute; *HW*, calculated heart weight; *EDD*, end diastolic diameter; *ESD*, end systolic diameter; *FS*, fractional shortening; *LVM*, left ventricular mass; *WTh*, wall thickness.

**Figure 2:**
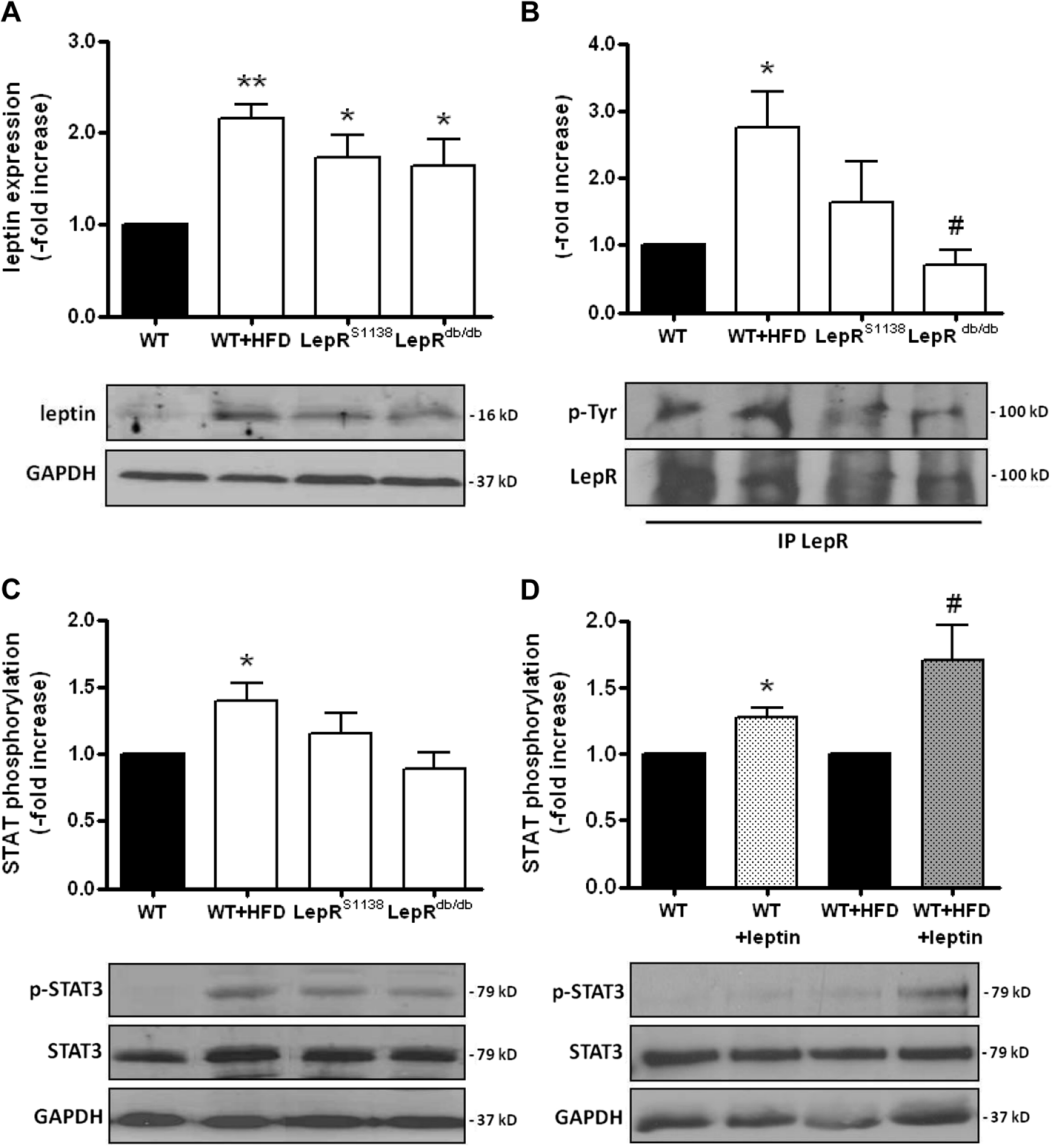
**Cardiac leptin expression and signal transduction in lean and obese mice.** Protein was extracted from hearts of 7 months-old mice (n = 8 per group) and analyzed for the expression of **(A)** leptin, **(B)** phosphorylated LepR (using immunoprecipitation of LepR, followed by the detection of total phosphotyrosines and LepR) and **(C)** phosphorylated STAT3. **(D)** Cardiac STAT3 phosphorylation in response (30 min later) to a single injection of recombinant murine leptin (1 mg/kg BW i.p.) was examined in WT (n = 4) and WT + HFD (n = 6) mice. Results are expressed as -fold increase of controls (black bars) after normalization for total protein and GAPDH expression. The mean ± SEM as well as representative Western blot results are shown. *P < 0.05 and **P < 0.01 vs. WT mice; #P < 0.05 vs. WT + HFD mice.

The presence of cardiac hypertrophy in LepR-deficient and, to a lesser extent also in diet-induced obese mice, suggests that it develops as a result of the heart’s inability to respond to elevated systemic (Table 1) and/or cardiac (Figure 2A) leptin levels. In this regard, Western blot analysis revealed increased levels of phosphorylated (p-) LepR (Figure 2B) and STAT3 (Figure 2C) protein in hearts of HFD-induced obese mice (P < 0.05 vs. WT for both), whereas findings in LepR^db/db^ mice did not differ from those in lean controls or were reduced compared to those in WT + HFD mice (P < 0.05 for differences in LepR phosphorylation). Moreover, both lean and HFD-induced obese WT mice responded to a single i.p. injection of recombinant murine leptin with a significant increase in the cardiac STAT3 phosphorylation (Figure 2D), suggesting a preserved cardiac leptin signal transduction in hyperleptinemic, diet-induced obese mice.

**Figure 3:**
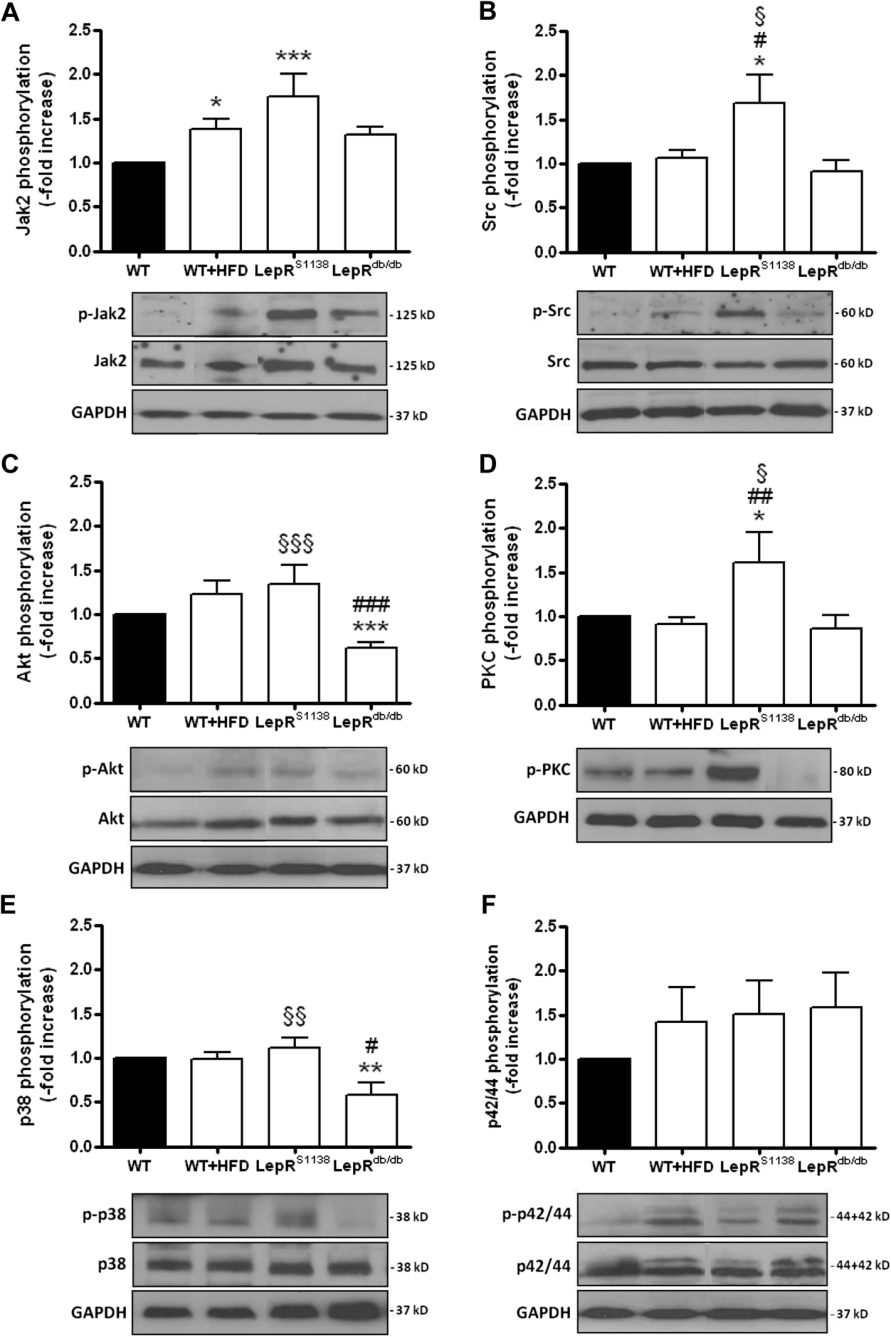
**Hypertrophic signal transduction in hearts of lean and obese mice.** Protein was isolated from hearts of 7 months-old WT (n = 15), WT + HFD (n = 12), LepR^S1138^ (n = 15) and LepR^db/db^ (n = 15) mice and analyzed for the expression of phosphorylated Jak2 **(A)**, Src kinase **(B)**, Akt **(C)**, PKC **(D)**, p38 **(E)** and p42/44 MAPK **(F)**. Results are expressed as -fold increase of lean control mice (after normalization for total protein [with the exception of PKC] and GAPDH expression). The mean ± SEM as well as representative findings are shown. *P < 0.05, **P < 0.01 and ***P < 0.001 vs. WT mice; #P < 0.05 and ##P < 0.01 vs. WT + HFD mice; §P < 0.05, §§P < 0.01 and §§§P < 0.001 for the difference between LepR^db/db^ and LepR^S1138^ mice.

To further study the role of leptin signaling in the development of cardiac hypertrophy and also to determine, whether the inability of leptin to activate STAT3 contributes to the cardiac maladaptation in obesity, we examined mice in which tyrosine (Tyr)1138 within LepR had been replaced by a serine (LepR^S1138^). In these mice, leptin cannot signal via STAT3, but continues to be able to activate Jak2 and SH2 domain-containing adapter proteins. Western blot analysis revealed that p-LepR (Figure 2B) and p-STAT3 (Figure 2C) levels in hearts of LepR^S1138^ mice did not significantly differ from those in WT and LepR^db/db^ mice. Similar to mice with complete LepR deficiency, lack of LepR-mediated STAT3 activation resulted in severe adiposity, although serum leptin levels were lower than those in LepR^db/db^ mice (P < 0.001; Table 1). Interestingly, obese LepR^S1138^ exhibited a more pronounced increase in mean heart weights not only compared to lean or diet-induced obese WT mice, but also compared to LepR^db/db^ mice (P < 0.001 for all comparisons; Table 1), and differences persisted after normalization for body weight (P < 0.001) or tibia length (P < 0.001). Echocardiography confirmed increased LV mass (P < 0.01) or heart weights (P < 0.001) in LepR^S1138^ mice compared to their LepR^db/db^ counterparts (Table 2; please also see Figure 1A-C). Moreover, hearts of LepR^S1138^ mice exhibited elevated levels of phosphorylated Jak2 (P < 0.001 vs. WT; Figure 3A), Src kinase (P < 0.05 vs. WT, WT + HFD and LepR^db/db^; Figure 3B), Akt (P < 0.001 vs. LepR^db/db^; Figure 3C), PKC (P < 0.05 vs. WT and LepR^db/db^, P < 0.01 vs. WT + HFD; Figure 3D) and p38 MAPK (P < 0.01 vs. LepR^db/db^; Figure 3E), suggesting that an intact, Tyr1138-independent LepR activation in the presence of elevated leptin levels may have contributed to the pronounced cardiac hypertrophy present in these mice. On the other hand, cardiac levels of p-p42/44 MAPK did not significantly differ between the mouse groups (Figure 3F).

**Figure 4:**
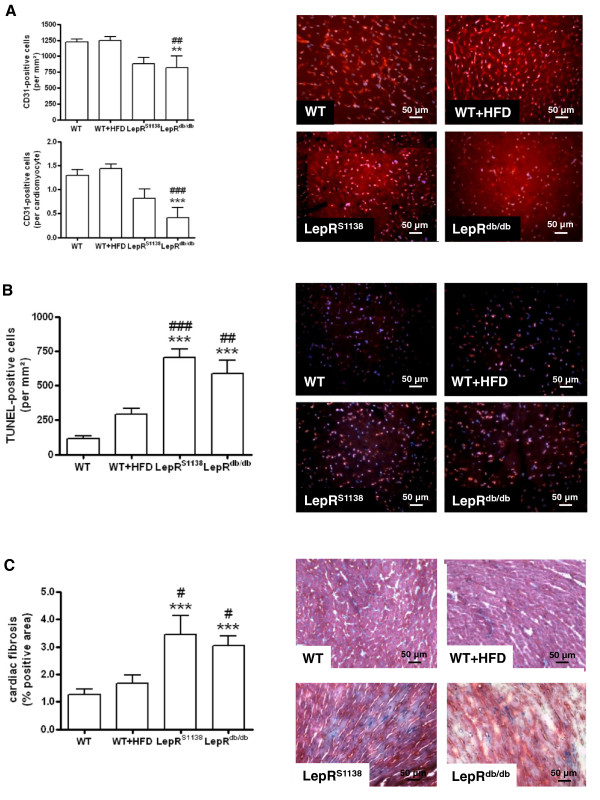
**Histological analysis of angiogenesis, apoptosis and fibrosis in hearts of lean and obese mice.** Serial cross sections through the LV of WT, WT + HFD, LepR^S1138^ and LepR^db/db^ mice (n = 10 per group) were immunostained and the number of **(A)** CD31-positive endothelial cells and **(B)** TUNEL-positive apoptotic cell nuclei determined. Results are expressed per cardiomyocyte and/or mm^2^. **(C)** The degree of cardiac fibrosis was quantified after Masson’s trichrome (MTC) staining. Results are expressed as % of total tissue area (at 200-fold magnification). The mean ± SEM as well as representative findings are shown. **P < 0.01 and ***P < 0.001 vs. WT; #P < 0.05, ##P < 0.01 and ###P < 0.001 vs. WT + HFD mice.

M-mode echocardiography also revealed significantly increased enddiastolic LV diameters in LepR^S1138^ mice (P < 0.01 vs. WT and LepR^db/db^ mice; Table 2; representative findings are shown in Figure 1B), suggesting that the observed (over-)activation of LepR signaling together with the inability to induce STAT3 may result in augmented hypertrophy and maladaptive cardiac remodeling. Of note, fractional shortening (FS) was not significantly altered in HFD-induced obese WT mice (P = n.s. vs. WT mice), but found to be increased in both LepR^db/db^ (P < 0.01 vs. WT and P < 0.001 vs. WT + HFD mice) and LepR^S1138^ mice (P < 0.05 vs. WT and P < 0.001 vs. WT + HFD mice). Histological analyses revealed significantly reduced numbers of CD31-positive capillary endothelial cells in LepR^db/db^, and to a lesser extent also in LepR^S1138^ mice (Figure 4A), whereas the number of TUNEL-positive apoptotic cells (Figure 4B) and the fibrotic tissue area (Figure 4C) were found to be significantly increased in hearts of both LepR^S1138^ and LepR^db/db^ mice compared to lean and diet-induced obese WT mice.

**Figure 5:**
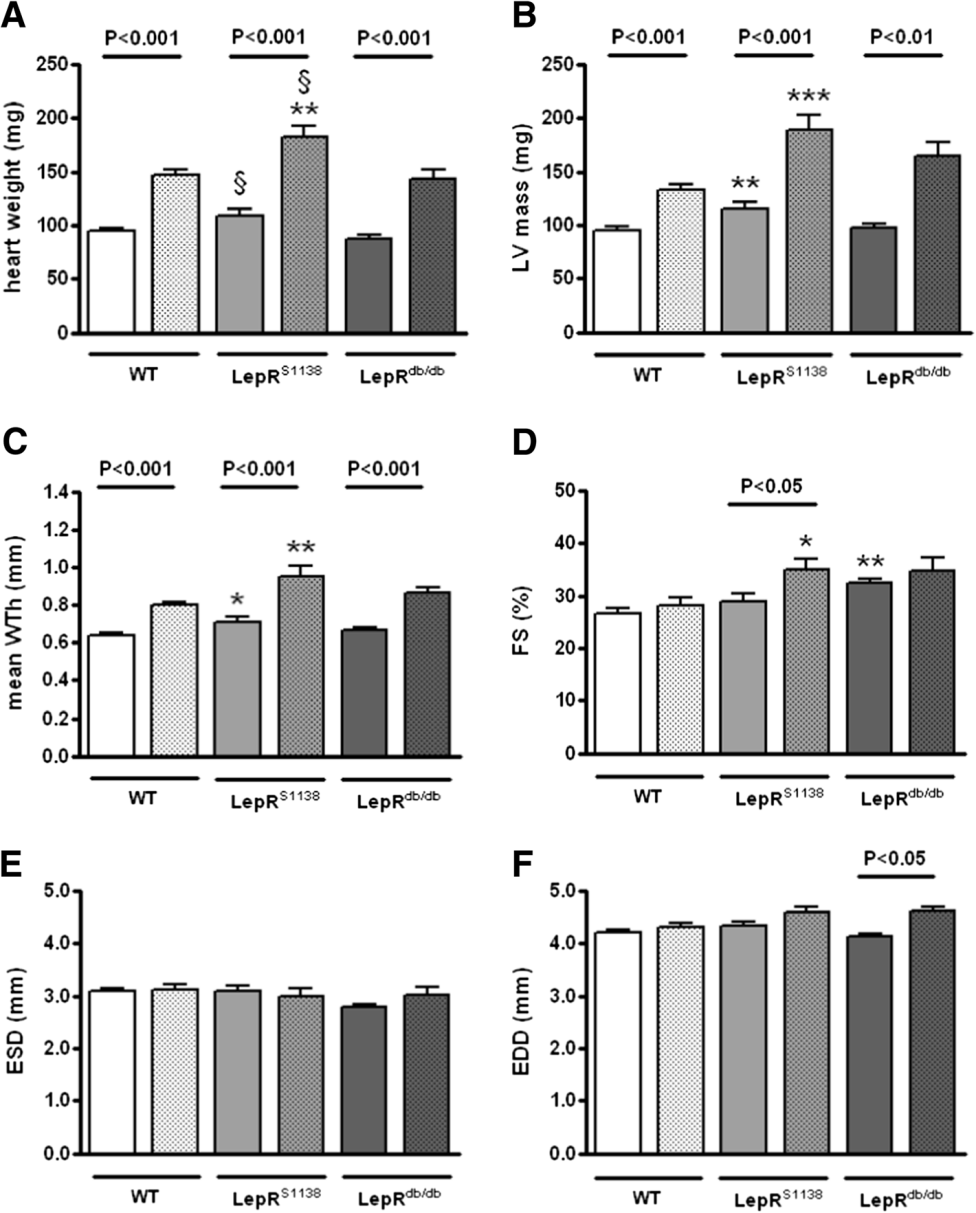
**Echocardiography findings in young lean and obese mice before and after chronic β-adrenergic stimulation.** Isoprenaline-filled osmotic minipumps were subcutaneously implanted into 2 months-old WT (n = 12), LepR^S1138^ (n = 9) and LepR^db/db^ (n = 7) mice to examine the cardiac response to a hypertrophic stimulus other than leptin. Echocardiography **(A-F)** was performed immediately before (open bars) as well as at the time of tissue harvest 14 days later (dotted bars). *P < 0.05, *P < 0.01 and ***P < 0.001 for differences vs. WT mice; §P < 0.05 for differences between LepR^db/db^ and LepR^S1138^ mice. Significance levels for differences before and after isoprenaline stimulation (as determined using Student’s *t* test for paired means) are indicated within the graph.

**Figure 6:**
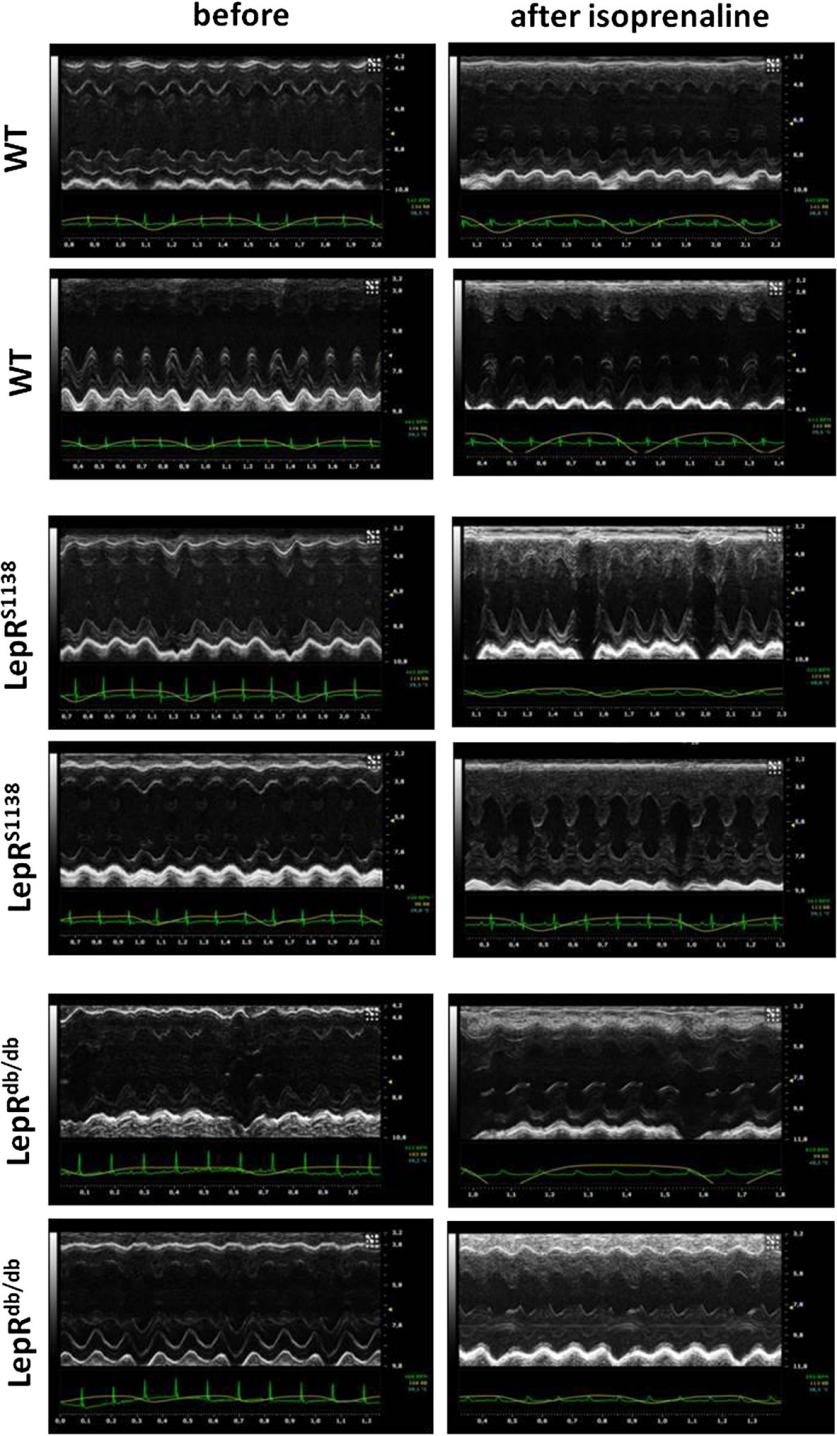
Representative M-mode echocardiography recordings.

To examine the specificity of leptin’s hypertrophic action in obesity, the cardiac response of young, i.e. 2 months-old WT (n = 12; body weight, 22 ± 0.9 g), LepR^S1138^ (n = 9; 34 ± 1.1 g, P < 0.001 vs. WT) and LepR^db/db^ mice (n = 7; 40 ± 1.3 g; P < 0.001 vs. WT and P < 0.01 vs. LepR^S1138^) to chronic isoprenaline infusion (20 mg/kg BW per day) was examined. Under basal conditions, similar findings as those in 7 months-old mice were observed, i.e. LepR^S1138^ mice exhibited an increased heart weight (P < 0.05 vs. LepR^db/db^; Figure 5A), LV mass (P < 0.01 vs. WT; Figure 5B) and mean WTh (P < 0.05 vs. WT; Figure 5C), whereas other changes, such as differences in fractional shortening (Figure 5D), ESD (Figure 5E) and EDD (Figure 5F) were not (yet) detected. On the other hand, all mouse groups responded to chronic β-adrenergic stimulation with significant cardiac hypertrophy, and no differences (with the exception of heart weight; Figure 5A) were observed between LepR^S1138^ and LepR^db/db^ mice. Representative M-mode echocardiography tracings are shown in Figure 6 and summarized in Additional file 2: Table S2.

## Discussion

The adipocytokine leptin may link obesity with cardiac hypertrophy, an important risk factor for the development of heart failure. Studies in humans [2,3] and rodents [10,11] have shown that obesity is associated with LV hypertrophy, and body mass index was identified as a strong and independent predictor of LV mass [2,3]. Importantly, cardiac hypertrophy is also observed in normotensive obese subjects [6], and plasma leptin levels are associated with increased myocardial wall thickness independent of BW or blood pressure elevations [5], suggesting a causal role for leptin in the pathogenesis of cardiac hypertrophy.

Although the major source of leptin is adipose tissue, cardiomyocytes are also capable of synthesizing leptin [27], and increased cardiac leptin levels have been reported in mice or rats following coronary ligation [13,18] or in patients with heart failure [28]. In this study, elevated circulating as well as cardiac leptin levels were detected in both diet-induced and genetically obese mice, which may have acted on cardiomyocytes as well as other, non-cardiomyocyte cells expressing leptin receptors [29]. Although leptin serum levels were higher than in previous publications [30], we explain this findings with the higher age of the mice, a factor previously found to be associated with increased circulating leptin levels [31]. Leptin has been shown to stimulate the hypertrophy of cardiomyocytes isolated from rats [7,20] or humans [8,19]. Moreover, chronic leptin infusion increased cardiac ANP expression after myocardial infarction (MI) in mice [32], whereas neutralizing LepR antibodies abrogated the hypertrophy of the surviving myocardium after coronary artery ligation in rats [33]. On the other hand and as confirmed in our analysis, cardiac hypertrophy also develops in leptin- and LepR-deficient mice and may be reversed by leptin substitution [12]. Caloric restriction experiments suggested that the anti-hypertrophic effects of leptin had occurred in addition to weight loss [12], which itself may preserve heart function and attenuate LV remodeling [34]. Thus, it is unclear whether the cardiac hypertrophy in obesity is the consequence of pro-hypertrophic effects of the adipokine or rather the result of a resistance towards leptin’s preventive effects on hypertrophic cardiac remodeling. Of note, since body weight is markedly elevated in the diet-induced and particularly, the genetically obese mice, the heart-to-body weight ratio decreases, even though the absolute heart weight is increased (but to a relatively lesser extent).

Obesity is associated with elevated circulating leptin levels and hypothalamic resistance to the weight-reducing effects of the adipokine, whereas the existence of a peripheral (e.g. cardiac) leptin resistance is controversial. For example, reduced cardiac LepR expression has been reported in HFD-fed rats [14], whereas others demonstrated unaltered cardiac STAT3 phosphorylation in diet-induced obese rodents following acute leptin administration [15-17]. Our findings also suggest that hearts from diet-induced obese mice continue to respond to leptin in the presence of chronically elevated leptin levels and that the observed elevation of serum and cardiac leptin may thus contribute to the development of cardiac hypertrophy in obesity. For example, hearts of hyperleptinemic obese WT mice (i.e. those with intact leptin receptors) exhibited signs of activated leptin signaling, including elevated levels of phosphorylated LepR and STAT3, while they were unchanged or reduced in mice with mutated or truncated forms of LepR (i.e. LepR^S1138^ or LepR^db/db^ mice). Moreover, both lean and obese WT mice responded to a single leptin injection with increased cardiac STAT3 phosphorylation. Of note, we could not spatially dissect the cardiac responsiveness to leptin, since whole heart homogenates were examined. Possible explanations underlying the discrepancy between the present and some previous studies include the animal species, as the absence of a response to leptin in obesity has been so far primarily observed in rats [14]. In addition, age, sex and feeding status of the animals or the time of recombinant leptin administration may have influenced the results. Of note, previous studies in humans have reported the existence of individuals (up to 40%) exhibiting a blunted response to leptin [35], although it is unknown, whether such phenomenon also occurs in rodents.

Interestingly, hearts from LepR^S1138^ mice exhibited a marked overactivation of STAT3-independent leptin signaling pathways, including Jak2, Src kinase, Akt or p38 MAPK, i.e. factors previously shown to mediate the pro-hypertrophic effects of the adipokine in cardiomyocytes [19,20]. Importantly, overactivation of leptin signaling in hearts of LepR^S1138^ mice was accompanied by a pronounced cardiac hypertrophy, both at the organ and the single cardiomyocyte level, despite similar adiposity. Although leptin levels were found to be lower in LepR^S1138^ compared to LepR^db/db^ mice, as previously reported [23], leptin continues to be able to activate LepR signal transduction in these mice, for example via LepR-Tyr985. Similar echocardiographical findings were obtained in young (i.e. 2 months-old) and older (i.e. 7 months-old) mice, arguing against the development of cardiac hypertrophy secondary to hemodynamic or other metabolic changes associated with obesity, although we cannot exclude the possible contribution of a more pronounced hyperinsulinemia [23] to the development of cardiac hypertrophy in LepR^S1138^ mice. On the other hand, hypertension had not been observed in ob/ob mice [12], and heart weight increase and concentric LV hypertrophy in obese mice and humans also occurs without systolic and diastolic blood pressure elevations [5,6,36].

Although a predominant cardiac expression of the short (i.e. without STAT3 binding site) over the long LepR isoform has been reported [7,29], previous studies have shown that stimulation of neonatal rat cardiomyocytes with leptin increased STAT3 phosphorylation, nuclear translocation and DNA binding activity [32]. Also, cardiac STAT3 activation after MI was blunted in leptin-deficient mice [13]. The observation that increased cardiac STAT3 phosphorylation in hyperleptinemic, diet-induced obese mice was reduced or almost completely abolished in LepR^S1138^ or LepR^db/db^ mice suggests that cardiac STAT3 activation in obesity largely occurs downstream of elevated leptin levels and that other cytokines, also elevated in obesity and known to signal via Jak2-STAT3, may be of minor importance. On the other hand, the importance of leptin-mediated STAT3 activation in the heart and its contribution to cardioprotective signaling pathways *in vivo* have not been directly examined so far.

STAT3 has been implicated in cardioprotection after various injuries. For example, cardiomyocyte-specific STAT3 deletion results in dilatative cardiomyopathy, characterized by increased apoptosis and interstitial fibrosis as well as reduced myocardial capillary density [21,22]. Previous studies suggested that leptin may exert beneficial effects on the heart. For example, administration of leptin was associated with smaller infarct size after ischemia/reperfusion injury [37], whereas ischemic postconditioning failed to induce cardioprotection in mice lacking leptin or its receptor [38]. Also, leptin deficiency was associated with a worsened cardiac function and survival after coronary artery ligation, which could be improved by leptin repletion [13]. Regarding possible mechanisms, increased cardiac myocyte apoptosis was observed in hearts from leptin (receptor)-deficient mice [39,40]. Similar findings were obtained *in vitro*, showing that leptin protects cardiomyocytes against apoptotic cell death induced by serum starvation [41]. Our analyses also revealed significantly elevated numbers of apoptotic cells in hearts of obese LepR^S1138^ and LepR^db/db^ mice, consistent with a reduced activation of STAT3-responsive anti-apoptotic genes [40]. Although findings in mice with systemic defects in leptin signal transduction may have been confounded by the concomitant presence of obesity and associated metabolic and inflammatory alterations, adverse cardiac remodeling after MI [42] or lethal heart failure [43] were recently reported in mice with cardiomyocyte-specific LepR deletion. On the other hand, the beneficial effects of leptin-mediated STAT3 activation may not be restricted to cardiomyocytes. For example, we and others have shown that leptin promotes the angiogenic properties of endothelial (progenitor) cells [25,44], and cardiac angiogenesis was reduced in LepR^S1138^ and LepR^db/db^ mice. In addition, hearts of obese LepR^S1138^ and LepR^db/db^ mice exhibited increased interstitial fibrosis, which may have occurred secondary to increased cardiomyocyte loss, although previous studies have shown that leptin may also directly influence myocardial matrix metabolism [45]. On the functional level, the enhanced activation of pro-hypertrophic signaling pathways in the absence of STAT3-mediated cardioprotection may have contributed to the echocardiographic finding of LV cavity dilation in LepR^S1138^ compared to LepR^db/db^ mice.

## Conclusions

Taken together, our findings suggest that hearts from diet-induced obese mice continue to respond to chronically elevated leptin levels and that increased systemic and/or local leptin and enhanced cardiac LepR activation contribute the development of cardiac hypertrophy. On the other hand, chronic overactivation of hypertrophic signaling mediators together with an inabilitity to activate STAT3-dependent cardioprotective pathways may promote maladaptive cardiac remodeling. Of note, our findings also indicate that leptin signaling is not a prerequisite to develop cardiac hypertrophy in obesity and that additional pathways also contribute to the increase in LV mass associated with higher body weight.

## Competing interests

The authors declare that they have no competing interests.

## Authors’ contributions

MLN and NMW carried out the protein and histological analyses of mouse hearts, performed the statistical analysis and drafted the manuscript. RG carried out and evaluated the TUNEL assays. MD aquired and analyzed the echocardiographical data. SK and GH participated in the design of the study and critically revised it for its contents. KS conceived and designed the study, wrote the manuscript, and aquired funding. All authors have read and approved the final manuscript.

## Supplementary Material

Additional file 1: Table S1 Diet composition. Click here for file

Additional file 2: Table S2 Echocardiographic parameter in 2 months-old mice before and 14 days after isoprenaline infusion. Click here for file
