# Supplementary material for: Importance of leptin signaling and signal transducer and activator of transcription-3 activation in mediating the cardiac hypertrophy associated with obesity
Source: J Transl Med. 2013 Jul 11;11:170. doi: 10.1186/1479-5876-11-170 (PMC3717024; doi:10.1186/1479-5876-11-170)
Supplement: Additional file 1: Table S1 — Diet composition. [file 1479-5876-11-170-S1.doc]

**Table S1. Diet c**omposition

|  | **high fat diet**  **(TD 12451)** | | **normal chow**  **(TD 12450B)** | |
| --- | --- | --- | --- | --- |
| **energy from** | | | | |
|  | ***g%*** | ***kcal%*** | ***g%*** | ***kcal%*** |
| **protein** | 24 | 20 | 19 | 20 |
| **carbohydrates** | 41 | 35 | 67 | 70 |
| **fat** | 24 | 45 | 4 | 10 |
| **ingredients** | | | | |
|  | ***g%*** | ***kcal*** | ***g%*** | ***kcal*** |
| **casein** | 200 | 800 | 200 | 800 |
| **L-cystine** | 3 | 12 | 3 | 12 |
| **corn starch** | 73 | 291 | 315 | 1,260 |
| **maltodextrin** | 100 | 400 | 35 | 140 |
| **sucrose** | 173 | 691 | 350 | 1,400 |
| **soybean oil** | 25 | 225 | 25 | 225 |
| **lard** | 178 | 1,598 | 20 | 180 |
| **energy content, *kcal/g*** | 4.73 | | 3.85 | |

Values according to the manufacturer’s (Research Diets Inc.) specifications.
