# Supplementary material for: Importance of leptin signaling and signal transducer and activator of transcription-3 activation in mediating the cardiac hypertrophy associated with obesity
Source: J Transl Med. 2013 Jul 11;11:170. doi: 10.1186/1479-5876-11-170 (PMC3717024; doi:10.1186/1479-5876-11-170)
Supplement: Additional file 2: Table S2 — Echocardiographic parameter in 2 months-old mice before and 14 days after isoprenaline infusion. [file 1479-5876-11-170-S2.docx]

**Table S2. Echocardiographic parameter in 2 months-old mice before and 14 days after isoprenaline infusion**

|  | WT | | LepR^S1138^ | | LepR^db/db^ | |
| --- | --- | --- | --- | --- | --- | --- |
| n | 12 | | 9 | | 7 | |
| heart rate  (bpm) | 448 ± 8.2 | 579 ± 11  ### | 461 ± 8.3 | 559 ± 20  ## | 454 ± 11 | 590 ± 16  ## |
| WTh  (mm) | 0.64 ± 0.01 | 0.80 ± 0.02  ### | 0.71 ± 0.03  * | 0.95 ± 0.06  ** ### | 0.67 ± 0.01 | 0.86 ± 0.03  ### |
| ESD  (mm) | 3.1 ± 0.1 | 3.1 ± 0.1 | 3.1 ± 0.1 | 3.0 ± 0.1 | 2.8 ± 0.1 | 3.0 ± 0.2 |
| EDD  (mm) | 4.2 ± 0.1 | 4.3 ± 0.1 | 4.3 ± 0.1 | 4.6 ± 0.1 | 4.1 ± 0.1 | 4.6 ± 0.1  # |
| LVM  (mg) | 95 ± 3.2 | 133 ± 4.9  ### | 116 ± 6.0  ** | 188 ± 15  *** ### | 98 ± 3.1 | 165 ± 13  ## |
| HW  (mg) | 95 ± 2.5 | 147 ± 4.7  ### | 108 ± 6.4  § | 183 ± 10  ** ### § | 87 ± 4.5 | 144 ± 9.0  ### |
| FS  (%) | 27 ± 0.8 | 28 ± 1.4 | 29 ± 1.6 | 35 ± 1.8  * # | 32 ± 1.0  ** | 35 ± 2.6 |

*P<0.05, *P<0.01 and ***P<0.001 for differences vs. WT mice; #P<0.05, ##P<0.01 and ###P<0.001 for differences before and after isoprenaline stimulation; §P<0.05 for the difference between LepR^S1138^ and LepR^db/db^ mice. Abbreviations: bpm, beats per minute; EDD, end diastolic diameter; ESD, end systolic diameter; FS, fractional shortening; HW, calculated heart weight; LVM, left ventricular mass; WTh, wall thickness.
